# Genome-wide transcriptome analysis reveals the molecular mechanism of high temperature-induced floral abortion in *Litchi chinensis*

**DOI:** 10.1186/s12864-019-5493-8

**Published:** 2019-02-11

**Authors:** Hao Liu, Congcong Wang, Houbin Chen, Biyan Zhou

**Affiliations:** Guangdong Litchi Engineering Research Center, College of Horticulture, South China Agricultural University, Guangzhou, 510642 China

**Keywords:** Flowering, Abortion, Transcriptome, Litchi, High temperature

## Abstract

**Background:**

Warm winter and hot spring attributed to global warming affected floral development and may induce floral abortion, resulted in poor flowering in litchi. To identify genes potentially involved in litchi floral abortion, six RNA-sequencing (RNA-Seq) libraries of the developing panicles (DPs) under low temperature (LT) conditions and the shrinking panicles (SPs) under high temperature (HT) conditions were constructed.

**Results:**

3.07–8.97 × 10^6^ clean reads were generated. Digital expression of the DPs with that of the SPs was compared. As a result, 1320 up-regulated and 981 down-regulated differentially expressed genes (DEGs) were identified. From the enriched GO-term, 54 temperature responsive DEGs, 23 hormone homeostasis- or biosynthesis-related DEGs, 137 hormone signal transduction or responsive DEGs, and 18 flowering-related DEGs were identified. Partial Least Squares Structural Equation Modeling (PLS-SEM) analysis indicated that the effects of hormone-related DEGs on *NAC*s, *MYB*s, *WRKY*s were stronger than that on flowering-related DEGs. Expression pattern analysis of the inflorescence in ‘Nuomici’ and ‘Huaizhi’ under LT and HT conditions showed that genes homologous to *AIL6* (*LcAIL6*), *LHY* (*LcLHY*), *MED16* (*LcMED16*), *SKIP20* (*LcSKIP20*), *POD20* (*LcPOD20*) in the two cultivars had similar expression trends.

**Conclusion:**

This study elucidated the transcriptome in the HT-induced floral abortion and identified key genes involved in the process. *NAC*s, *MYB*s, *WRKY*s may act as central players involved in the HT-induced floral abortion underlying hormonal control. Increased transcript in *LcLHY*, *LcMED16*, *LcSKIP20*, *LcPOD20* and decreased transcript in *LcAIL6* might be related to the inhibition of floral development. Our studies provided potential genes for the future molecular breeding of new cultivars that can reduce floral abortion under warm climates, and a novel clue to reveal the relationship of biological processes based on the RNA-seq data using PLS-SEM.

**Electronic supplementary material:**

The online version of this article (10.1186/s12864-019-5493-8) contains supplementary material, which is available to authorized users.

## Background

Litchi is an evergreen woody tree cultivated in subtropical and tropical regions and produces arillate fruits with sweet, translucent, juicy flesh [1]. However, unstable flowering causing irregular bearing affects the litchi industry. Up till now, many studies indicated that chilling is an irreplaceable factor for litchi flowering [2, 3]. In winter, litchi trees are subjected to low temperature for floral induction. After winter temperature treatment, litchi floral buds which are a mix of axillary and apical panicle primordia, leaf primordia or rudimentary leaves, break and the panicle primordia start to develop [4]. At this time point, whether panicle primordia can develop continually depends on environmental temperature. If the temperature is still low enough, the panicle primordia develop, and the rudimentary leaves cease growing and abscise automatically. The mixed buds will develop into pure/high-quality panicles [5]. However, warm winter and hot spring attributed to global warming frequently happen. The rudimentary leaves may develop into fully expanded leaves (panicle leaves), and the panicle primordia stop developing and shrink [6]. These mixed buds may become leafy panicles or even vegetative shoots, resulting in poor flowering [5]. How to control panicle leaf growth and inhibit floral abortion is of great importance to litchi production. In practice, ethephon is normally used to control panicle leaf growth under high-temperature conditions [7]. Methyl viologen dichloride hydrate, a reactive oxygen species (ROS) producer, and sodium nitroprusside, a nitric oxide (NO) donor are also proved to effectively inhibit panicle leaf growth and floral abortion [5]. Therefore, understanding the regulatory mechanism of floral abortion and panicle leaf controlled by low temperature or by chemicals is of great importance for flowering regulation in litchi.

We have previously focused on the feature of the low temperature-, ROS-, and NO-induced senescence of panicle leaves, and found that programmed cell death (PCD) is involved in the panicle leaf growth regulation by ROS and NO, as well as by low temperature in litchi [6, 8]. We also screened PCD related genes from our RNA-seq data sets of the ROS-treated rudimentary leaves [9], and identified a litchi homolog *MCII* (*LcMCII-1*). Silencing *LcMCII-1* by virus-induced gene silencing (VIGS) delayed ROS-dependent senescence. Ectopic over-expression of *LcMCII-1* in transgenic *Arabidopsis* promoted ROS-dependent and natural senescence [10], suggesting that *LcMCII-1* is positively involved in the regulation of rudimentary leaf senescence in litchi. These results provide a new target for the future molecular breeding of new cultivars that can produce high-quality flowers in warmer climates. However, other than studies on panicle leaf control, how the panicle primordia abort or stop developing under high-temperature conditions is still poorly known.

RNA sequencing (RNA-seq) technology is a powerful tool for plant studies. It has been used to identify key candidate genes in response to vernalization of oriental lily, and MADS-box family genes related to organ development and stress resistance in *Brassica rapa* [11, 12], compare transcriptome between low- and high-cadmium-accumulating *Brassica chinensis* genotypes in response to cadmium stress [13], reveal co-expression networks of transcription factors and wood component genes in *Populus trichocarpa* [14].

In this study, we focused on the molecular mechanism of floral abortion under high-temperature conditions. We used RNA-Seq technology to compare transcriptomes between the shrinking panicles (SPs) and the developing panicles (DPs). For RNA-Seq library construction, we used ‘Nuomici’ litchi as it is sensitive to HT in which floral buds easily cease to develop, and the panicle leaves growth vigorously according to our previous studies [4, 6], so that we could get more aborted inflorescences for RNA-seq. The litchi potted trees of ‘Nuomici’ were transferred to growth chambers for low temperature (LT) treatment to promote panicle/floral development and high-temperature treatment (HT) to induce panicle/floral abortion. The expression of the DPs and SPs was compared to identify differentially expressed genes (DEGs) potentially involved in litchi floral abortion. Then the expression pattern of the candidate genes in different cultivars under HT or LT conditions were determined. Partial Least Squares Structural Equation Modeling (PLS-SEM) analysis was also performed to quantify the relationship among the biological processes based on the transcript levels of the DEGs. We aim to reveal the molecular mechanism of the floral abortion, and to provide potential genes for the future molecular breeding of new cultivars that can set fruit in warmer climates.

## Results

### Morphology of DPs and SPs

**Fig. 1:**
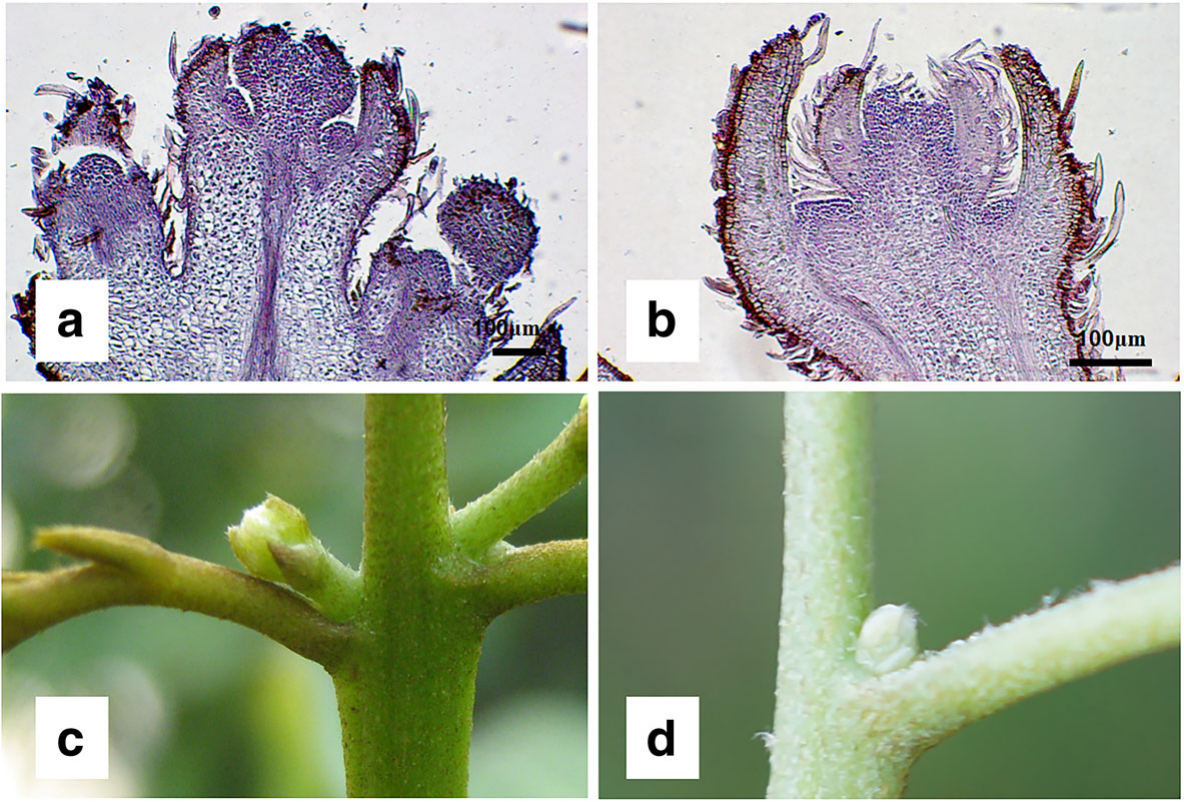
Morphology of the panicles showing a developing panicle (DP) in low temperature (**a**, **c**) and a shrinking panicle (SP) in high temperature (**b**, **d**)

To identify genes involved in floral abortion, ‘Nuomici’ trees were grown under HT to induce floral abortion and to produce SPs, or LT to promote floral development and produce DPs as a control. As a result, the percentage of flowering terminal shoots of the HT-treated trees (50.1%) was significantly lower than that of the LT-treated trees (85.3%) at 0.05 probability level. Figure 1 shows the morphology of a DP in LT and an SP in HT. The DP shows a main-order axis and two second-order axes in the inflorescence. Differentiation of axillary panicles progresses basipetally, showing the activity of the apical meristems, while the SP shows a delay or cease of development, indicating a low activity of meristem.

### Digital transcriptome analysis and DEGs identification

Six RNA-seq. libraries of DP and SP were constructed to identify genes potentially involved in HT-induced floral abortion. As shown in Table 1, we have generated 3.12–9.27 × 10^6^ raw reads and got 3.07–8.97 × 10^6^ clean reads from the libraries, with clean read ratios more than 95%. All the clean reads were mapped to the litchi transcriptome with mapping rates more than 55%. As a result, we obtain 1.91–5.24 × 10^6^ mapped reads for the six libraries.

**Table 1:** Throughput and quality of RNA-seq of the litchi floral buds

Treatments	Raw reads	Clean reads	Clean reads/ raw reads (%)	Unique match reads	Mapping rate (%)
DP1	9,265,430	8,966,317	96.77	5,239,915	58.44%
DP2	6,289,671	6,000,114	95.40	3,304,262	55.07%
DP3	3,119,925	3,073,896	98.52	1,908,274	62.08%
SP1	7,928,755	7,776,894	98.08	4,413,387	56.75%
SP2	5,887,516	5,814,604	98.76	3,428,290	58.96%
SP3	8,117,449	7,922,273	97.60	4,738,311	59.81%

‘Nuomici’ litchi trees with panicle primordia were transferred to a growth chamber at 12-h photoperiod with a temperature of 18 °C (LT) to encourage panicle development, or a growth chamber at the same photoperiod with a temperature of 26 °C (HT) to induced abortion of floral buds. The treatments in the first column of the table indicate three replicates of DP and SP, respectively

The unique match reads from the 6 libraries were remapped to the reference sequences and the unigene expression was normalized to FPKM. A linear regression analysis result of the fold-change in the gene expression ratios between RNA-seq and qRT-PCR was performed. A significantly positive correlation was found between RNA-seq data and qRT-PCR data, suggesting a reliable transcriptome analysis using RNA-seq (Additional file 1: Figure S1).

**Fig. 2:**
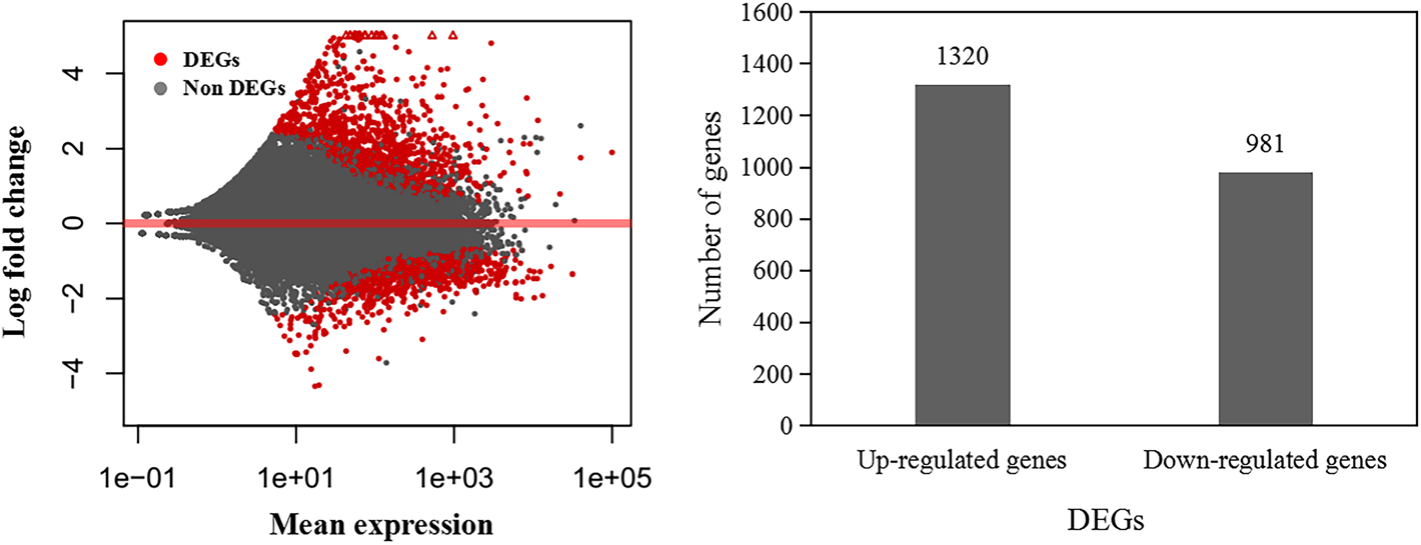
Analysis of the differentially expressed genes (DGEs) between developing panicle (DP) and shrinking panicle (SP). Each dot represents a gene. The x-axis represents the mean expression of the compared genes. The y-axis is the logarithm with base 2 of the fold change of the compared genes. Red dots represent DEGs

Correlation analysis of the DP and SP data suggested high repeatability of the sequencing samples (Additional file 2: Figure S2). DEGs between DP and SP were detected by DESeq2. As shown in Fig. 2, from the DEGs of the DP to those of the SP, 1320 up-regulated genes and 981 down-regulated genes were identified.

### GO-term analysis of DEGs

GO-term analysis was performed among the DEGs. The top 10 enriched GO terms are shown in (Additional file 3: Table S1). All the DEGs were classified into the cellular component, biological process, and molecular function. Under the biological process category, ribosome assembly, mature ribosome assembly, and ribonucleo protein complex assembly were the top abundant subcategories. Under cellular component, cytosolic part, cytosolic ribosome, and ribosomal subunit were the top abundant subcategories. For molecular function category, structural constituent of ribosome, structural molecule activity, and copper ion binding were the top abundant subcategories. Ribosome assembly related terms were among the most significantly enriched ones (Additional file 3: Table S1). Intriguingly, as shown in Table 2, several temperature-, hormone-, and flowering-related terms such as response to temperature stimulus, response to hormone, and regulation of floral meristem growth were significantly enriched in the biological process. Given their direct link to the variable we used and the physiological distinctions we observed in our experiment, we focused on those terms in our downstream analyses.

**Table 2:** Enriched biological process potentially involved in high temperature-induced floral abortion in litchi

Term	Term description	No. of genes	Frequence	*P*-value
GO:0010252	Auxin homeostasis	7	0.7%	0.00189
GO:0009409	Response to cold	37	3.9%	0.0051
GO:0048574	Long-day photoperiodism, flowering	6	0.6%	0.006
GO:0016131	Brassinosteroid metabolic process	9	1.0%	0.0065
GO:0009862	Systemic acquired resistance, salicylic acid mediated signaling pathway	6	0.2%	0.0074
GO:0009751	Phytosteroid biosynthetic process	8	0.9%	0.0095
GO:0009266	Response to temperature stimulus	52	5.5%	0.0104
GO:0006694	Steroid biosynthetic process	10	1.1%	0.013
GO:0010080	Regulation of floral meristem growth	2	0.2%	0.0136
GO:0010081	Regulation of inflorescence meristem growth	2	0.2%	0.0136
GO:0080142	Regulation of salicylic acid biosynthetic process	3	0.3%	0.0141
GO:0009725	Response to hormone	125	13.3%	0.0151
GO:0009808	Lignin metabolic process	9	1.0%	0.017
GO:0009753	Response to jasmonic acid	26	2.8%	0.0223
GO:0010451	Floral meristem growth	2	0.2%	0.0259
GO:0016132	Brassinosteroid biosynthetic process	7	0.7%	0.0264
GO:0010268	Brassinosteroid homeostasis	7	0.7%	0.0264
GO:0009697	Salicylic acid biosynthetic process	3	0.3%	0.0272
GO:0009737	Response to abscisic acid	59	6.3%	0.0272
GO:0009631	Cold acclimation	6	0.6%	0.0307
GO:0009735	Response to cytokinin	22	2.3%	0.0424

### Identification of flowering related genes involved in HT-induced floral abortion

**Fig. 3:**
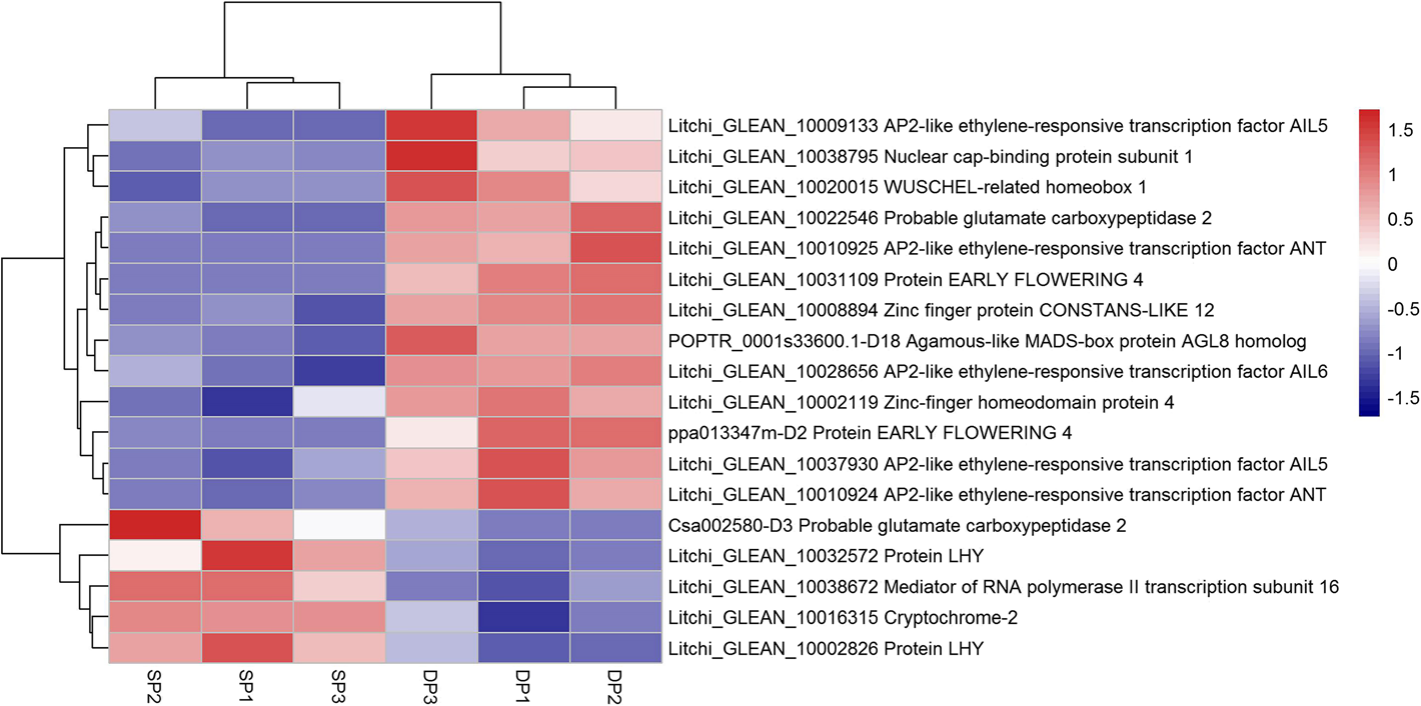
Heat map diagram showing the expression profiles of the flowering related differentially expressed genes (DEGs). Litchi trees when panicle primordia emerged were transferred to a growth chamber at 12-h photoperiod with a temperature of 18 °C (LT) to encourage floral development. The other trees were transferred to a growth chamber at 12-h photoperiod with a temperature of 26 °C (HT) to induced floral abortion. FPKM values of the developing panicle (DP) and the shrinking panicle (SP) were normalized to Z-score

From the enriched flowering related GO-term including regulation of floral meristem growth, regulation of inflorescence meristem growth, floral meristem growth, long-day photoperiodism (Table 2), we identified 8 flowering-related DEGs (Fig. 3). They encode homologous proteins including one Zinc-finger homeodomain protein (*ZHD4*), two Probable Glutamate Carboxypeptidase 2 (*AMP1*), one Nuclear Cap-binding Protein Subunit (*ABH1*), one Mediator of RNA Polymerase II Transcription Subunit 16 (*MED16*), two Late Elongated Hypocotyls (*LHY*), and one Cryptochrome-2 (*CRY2*). We also identified 10 other flowering related genes from the DEGs, such as Agamous-like MADS-box protein AGL8 encoding gene (*AGL8*), Protein EARLY FLOWERING 4 encoding gene (*ELF4*), Zinc finger protein CONSTANS-LIKE 12 encoding gene (*COL12*), and AP2-like ethylene-responsive transcription factor AINTEGUMENTA encoding gene (*ANT*). Compared to the DP, most of the flowering-related genes in the SP show down-regulated trends (13/18), such as the *AINTEGUMENTA-like AIL5* and *AIL6*, *AGL8*, *COL12*, and *ANT* (Fig. 3).

### Identification of the plant hormone-related genes involved in HT-induced floral abortion

**Fig. 4:**
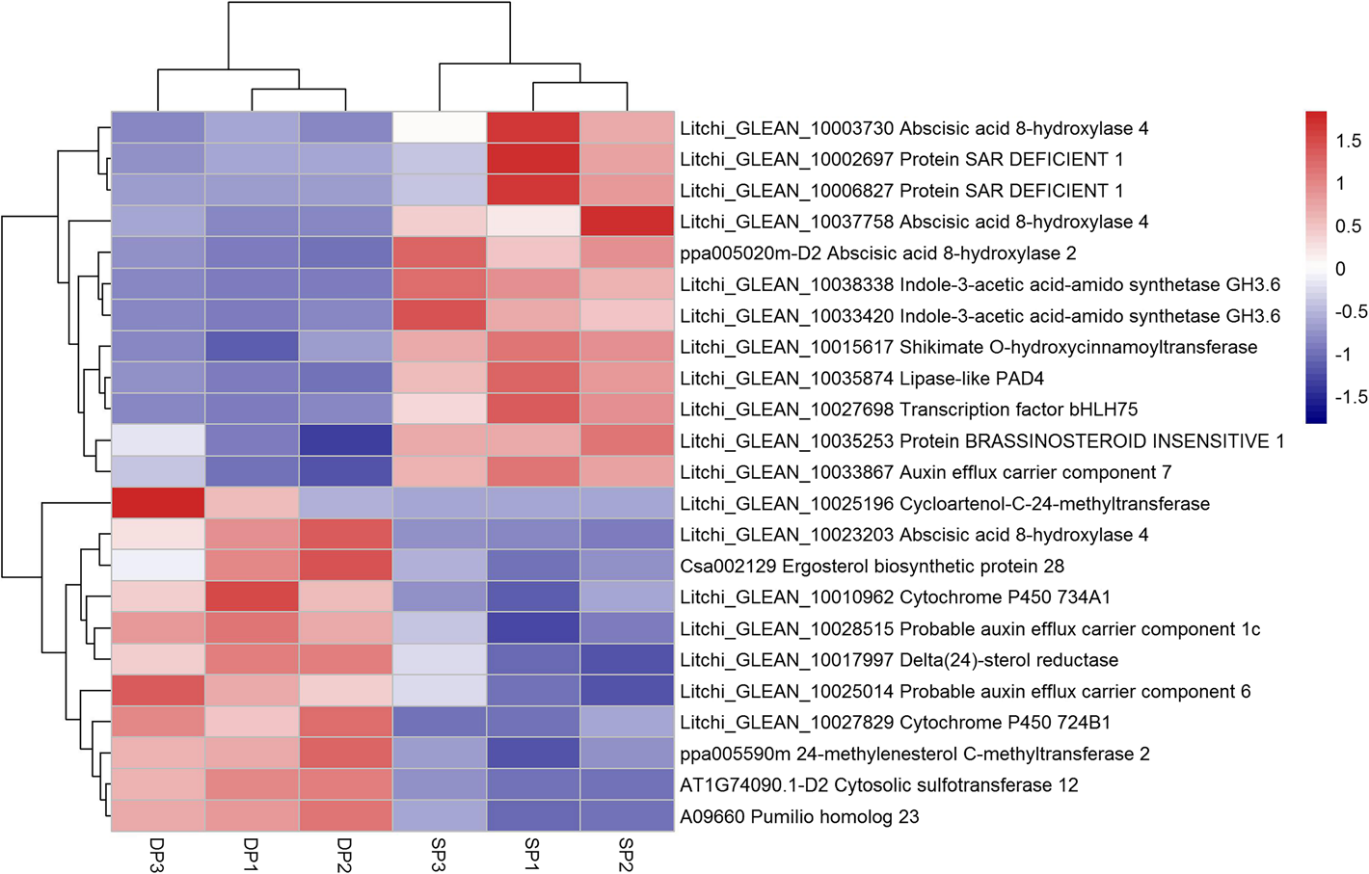
Heat map diagram showing the expression profiles of the hormone homeostasis related differentially expressed genes (DEGs). Litchi trees when panicle primordia emerged were transferred to a growth chamber at 12-h photoperiod with a temperature of 18 °C (LT) to encourage panicle development. The other trees were transferred to a growth chamber at 12-h photoperiod with a temperature of 26 °C (HT) to induced floral abortion. FPKM values of the developing panicle (DP) and the shrinking panicle (SP) were normalized to Z-score

From the enriched GO-term related to the control of plant hormone levels, we identified 23 DEGs that might control the hormone levels under different temperature conditions (Fig. 4). These enriched GO-terms are the auxin homeostasis, brassinosteroid metabolic process, steroid biosynthetic process, phytosteroid biosynthetic process, brassinosteroid biosynthetic process, brassinosteroid homeostasis, regulation of salicylic acid biosynthetic process, salicylic acid biosynthetic process (Table 2). The DEGs might be involved in hormone homeostasis, such as the Indole-3-acetic acid-amido synthetase GH3.6 encoding genes (*GH3.6*) that can prevent free IAA accumulation. They also might be involved in hormone biosynthesis process, such as the Delta (24)-sterol reductase encoding gene *DIM*, or they might control the hormone biosynthesis process, such as the Lipase-like PAD4 (PAD4) encoding gene (Fig. 4).

From the enriched plant hormone-related GO-term, we identified 137 plant hormone signaling or hormone responsive DEGs (Additional file 4: Figure S3; Additional file 5: Figure S4). These enriched GO-terms are salicylic acid mediated signaling pathway, response to jasmonic acid, response to abscisic acid, response to cytokinin, and response to hormone (Table 2). The DEGs encode proteins such as WRKY transcription factor (WRKY), Transcription factor MYB108 (MYB108), transcription factor MYB32 (MYB32), NAC transcription factor 56 (NAC056), Ethylene-responsive transcription factor (RAP2–3), Homeobox-leucine zipper protein (ATHB-7). Compared to the DP (from DP to SP), most of the hormone-controlled transcription factor encoding DEGs were up-regulated (Additional file 4: Figure S3). On the whole, more up-regulated DEGs were found in the enriched plant hormone-related GO-term (Additional file 4: Figure S3; Additional file 5: Figure S4).

### Pathway probably involved in the HT-induced floral abortion underlying hormonal control

**Fig. 5:**
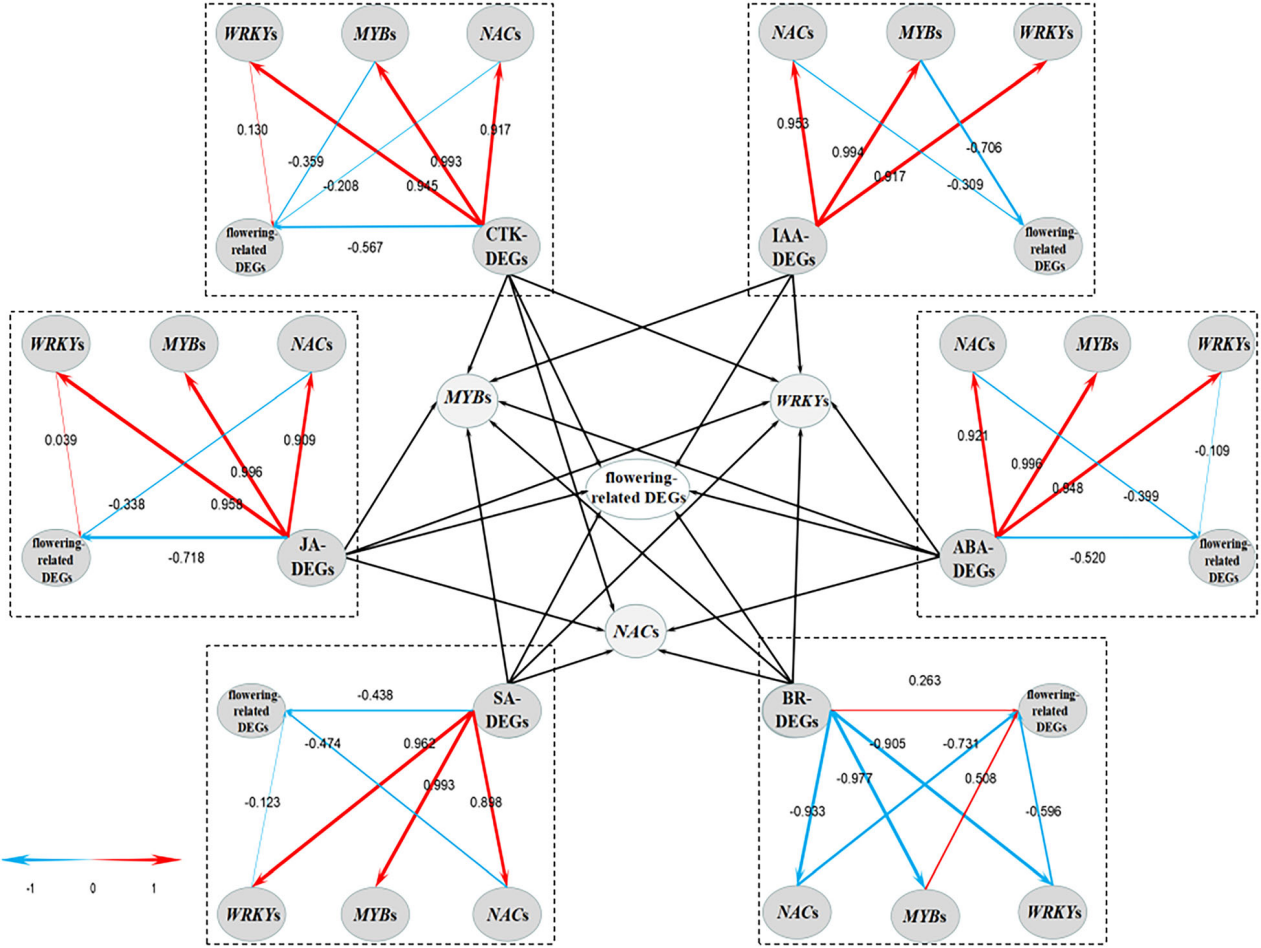
Direct graph of the Partial Least Squares structural equation modeling (PLS-SEM). The center part of the graph is based on the other six parts around. Levels of the path coefficients are reflected by the width of the red or blue arrows which indicate positive and negative effects, respectively. Values on the arrow indicate the intensity of the factor loading for the pathway. Pathways from hormone related DEGs to transcription factor encoding DEGs are first orders while those from transcription factor encoding DEGs to flowering related DEGs are second orders

To reveal a frame work of the HT-induced floral abortion by the regulation of hormone, we performed PLS-SEM analysis using the transcriptome data of the hormone-related and flowering-related DEGs as described above, and the main hormone-responsive transcription factor encoding DEGs including *NAC*s, *MYB*s, and *WRKY*s. We choose PLS for data analysis because it not only works well on removing the collinearity among variables but also works steadily with many kinds of database as well. In PLS-SEM, most of the latent variables were significant, outer model blocks have made an adequate explanation for latent variables (Additional file 6: Table S2). Average variance extracted (AVE, an indicator for converge validity) and composite reliability (indicator for internal consistency reliability) were higher than 0.5 and 0.7 (Additional file 7: Table S3), respectively, indicating that the model was reliable [15]. As shown in Fig. 5, there are two kinds of pathways for each model, the direct pathway and indirect pathway. In indirect pathways, the effects of hormone-related DEGs on the three main transcription factors and the effects of these transcription factors on flowering-related DEGs were positive or negative. All of them had a high factor loading such as 0.945, 0.993, and 0.917 in the CTK model, higher than the second order in indirect pathways (0.130, − 0.359 and − 0.208). Apart from IAA-related DEGs, the CTK-, JA-, SA-, BR-related DEGs were connected with flowering-related DEGs directly. Though IAA-related DEGs were not related to flowering-related DEGs directly, they were related to these DEGs through *NAC*s and *MYB*s. On the whole, the effects of hormone-related DEGs on *NAC*s, *MYB*s, *WRKY*s were stronger than that on flowering-related DEGs.

### Identification of temperature responsive genes involved in HT-induced floral abortion

**Fig. 6:**
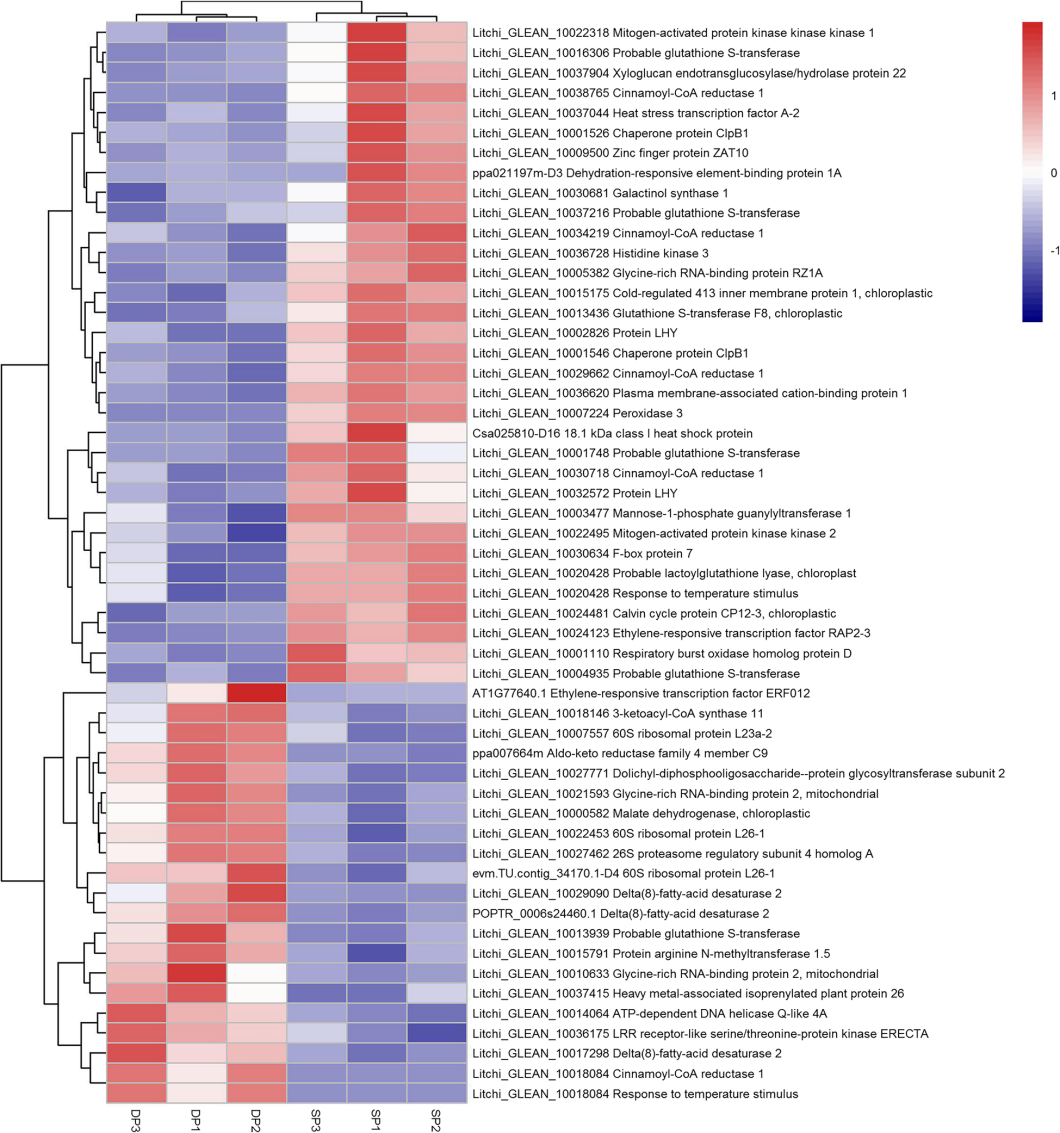
Heat map diagram showing the gene expression profiles of the temperature responsive differentially expressed genes (DEGs). Litchi trees when panicle primordia emerged were transferred to a growth chamber at 12-h photoperiod with a day/night temperature of 18 °C (LT) to encourage panicle development. The other trees were transferred to a growth chamber at 12-h photoperiod with a temperature of 26 °C (HT) to induced floral abortion. FPKM values of the developing panicle (DP) and the shrinking panicle (SP) were normalized to Z-score

From the enriched temperature-related GO-term including response to temperature stimulus, response to cold, cold acclimation, we identified 54 DEGs potentially involved in the HT-induced floral abortion. They encode homologous proteins such as Delta (8)-fatty-acid Desaturase 2 (SLD2), Cold-regulated 413 Inner Membrane Protein 1 (COR413IM1), Mannose-1-phosphate Guanylyltransferase (CYT1), Heat Stress Transcription Factor (HSFA2), Galactinol Synthase 1 (GOLS1), Dehydration-responsive Element-binding Protein 1A (DREB1A), Peroxidase 3 (PER3). Compared to the DP, most of the temperature-related genes of the SP showed up-regulated trends (33/54) (Fig. 6), such as the PER3, Heat stress transcription factor A-2 (HSFA2), Dehydration-responsive element-binding protein 1A (DREB1A) encoding genes. Some temperature responsive genes showed down-regulated trends, such as the Delta(8)-fatty-acid desaturase 2 (SLD2), Glycine-rich RNA-binding protein (RZ1A), and Probable glutathione S-transferase (HSP26-A).

### Expression profiles of the candidate genes in different cultivars under HT and LT conditions

To study detail expression patterns of the candidate genes in DPs and SPs, expression patterns of the candidate genes in the two widely cultivated ‘Nuomici’ and ‘Huaizhi’ under HT and LT conditions were studied. Flowering conditions of ‘Nuomici’ and ‘Huaizhi’ are shown in Table 3. Percentage of flowering terminal shoots in the LT-treated ‘Nuomici’ and ‘Huaizhi’ trees was significantly higher than that of the HT-treated trees. However, under HT condition, the percentage of flowering terminal shoots in ‘Huaizhi’ was higher than that in ‘Nuomici’. As to the percentage of leafy panicles, that of the LT-treated ‘Nuomici’ was the lowest, while that of the HT-treated ‘Nuomici’ was the highest. Compared to LT-treated trees, HT-treated trees of the two cultivars had smaller panicles according to the length of panicles and the length of axillary panicles, indicating that LT inhibited flowering.

**Table 3:** Effects of low temperature (LT) and high temperature (HT) on flowering of ‘Nuomici’ and ‘Huaizhi’ litchi trees

Flowering condition	‘Nuomici’		‘Huaizhi’	
	HT	LT	HT	LT
Percentage of flowering terminal shoots (%)	26.16 ± 1.36 a	67.66 ± 2.2 c	50.31 ± 3.62 b	62.86 ± 3.59 c
Percentage of leafy panicles (%)	70.61 ± 2.82 c	4.39 ± 1.65 a	37.17 ± 9.21 b	21.11 ± 4.88 ab
Length of panicles (cm)	11.98 ± 0.06 a	16.78 ± 0.26 c	13.49 ± 0.42 b	16.60 ± 0.19 c
Length of axillary panicles (cm)	4.72 ± 0.28 a	13.86 ± 0.34 a	5.938 ± 0.34 b	14.08 ± 0.24 b

**Fig. 7:**
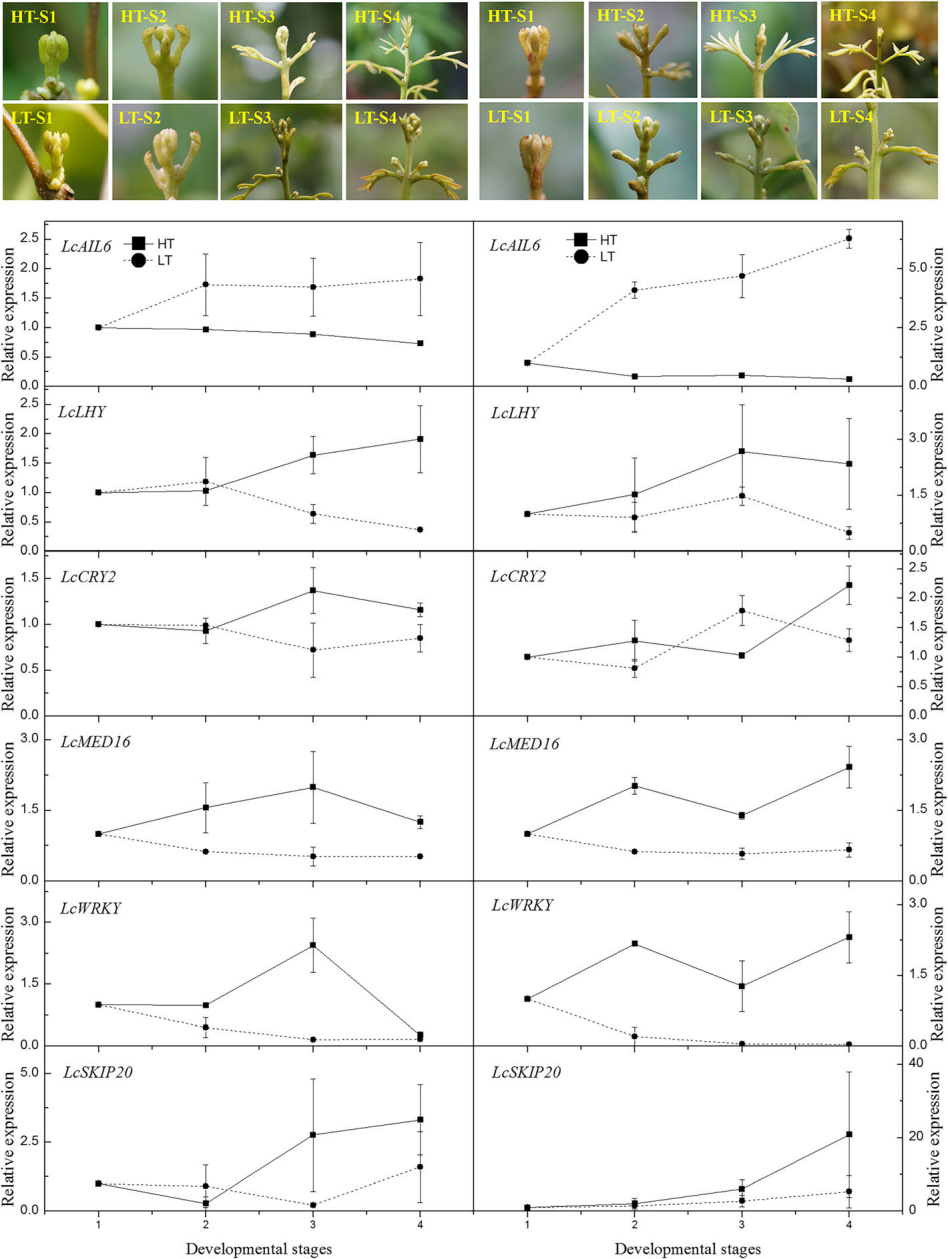
Expression profiles of the candidate genes of panicles in ‘Nuomici’ (left side) and ‘Huaizhi’ (right side) litchi trees under high temperature (HT) and low temperature (LT) conditions. ‘Nuonici’ and ‘Huaizhi’ trees at the stage of panicle primordia emergency were transferred to HT and LT growth chambers. Relative transcription was calculated by qRT-PCR using the 2^-ΔΔCT^ method with actin as a reference. Data are means of three replicates, and the bars represent SEs. Developmental stages 1 to 4 represent the panicle development stages. HT-S1 to HT-S4 represent primary stage, enlarging stage, shrinking stage, and shrunk stage under HT condition. LT-S1 to LT-S4 indicate primary stage, enlarging stage, early elongating stage, and late elongating stage under LT condition

**Fig. 8:**
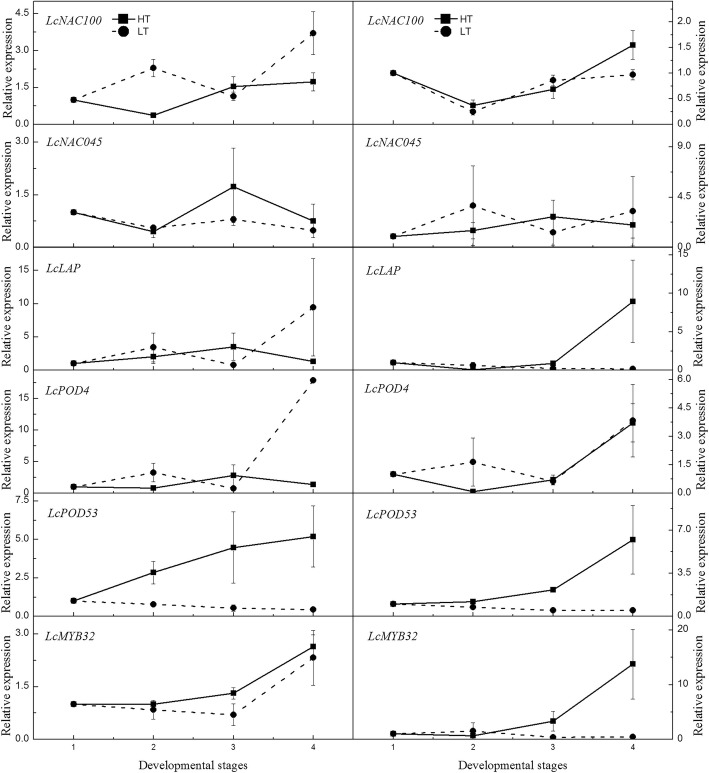
Expression profiles of the candidate genes of panicles in ‘Nuomici’ (left side) and ‘Huaizhi’ (right side) litchi trees under high temperature (HT) and low temperature (LT) conditions. ‘Nuonici’ and ‘Huaizhi’ trees at the stage of panicle primordia emergency were transferred to HT and LT growth chambers. Relative transcription was calculated by qRT-PCR using the 2^-ΔΔCT^ method with actin as a reference. Data are means of three replicates, and the bars represent SE. Developmental stages 1 to 4 represent the panicle development stages

Twelve genes identified from the flowering-, hormone-, and temperature response-related DEGs were selected. The expression pattern of the inflorescence in ‘Nuomici’ and ‘Huaizhi’ at the four panicle developmental stages under HT and LT (stages 1–4) according to Yang et al. [4] were determined. As shown in Figs. 7 and 8, the expression patterns of the genes homologous to *AIL6* (*LcAIL6*) in the two cultivars are similar. Those in LT showed increasing trends, while in HT showed decreasing trends and remained at lower levels, showing positive correlation to flowering. Genes homologous to *LHY* (*LcLHY*), *MED16* (*LcMED16*), *SKIP20* (*LcSKIP20*), *POD20* (*LcPOD20*) in the two cultivars showed similar expression trends. Those in HT showed increasing trends, while those in LT showed stable and remained at lower levels, showing a negative correlation to flowering. Similar expression patterns were found in *LcCRY2* in ‘Nuomici’, *LcWRKY*, *LcLAP*, and *LcMYB32* in ‘Huaizhi’. In ‘Nuomici’, floral buds under HT condition had relatively higher levels of *LcNAC045* and *LcMYB32* expression. However, no matter in ‘Nuomici’ or ‘Huaizhi’, *LcNAC100* and *LcPOD4* did not show any regular trends. On the whole, 5 out of the 12 candidate genes showed similar expression trends in the two cultivars and were correlated with flowering, and another 5 candidate genes in ‘Nuomici’ or ‘Huaizhi’ showed a similar relationship with flowering.

## Discussion

Warm winter always encourages vegetative growth and repress flowering formation of the evergreen fruit trees in tropical and subtropical areas. Litchi is an evergreen fruit tree whose floral induction and differentiation occur in winter and early spring. There exists competition between reproductive growth and vegetative growth in litchi similar to that of other evergreen fruit trees. Litchi floral buds consist of apical panicle primordia, axillary panicle primordia, and rudimentary leaves. These rudimentary leaves can expand quickly under high-temperature conditions, and then the panicle primordia stop developing and shrink. As a result, the floral buds develop to leafy panicles with few flowers or even reverse to vegetative shoots. Therefore, understanding the regulatory mechanism of floral abortion is as important as that of the transformation from vegetative meristems (VM) to inflorescence meristems (IM) for flowering regulation in litchi. In our previous study, we performed RNA-seq and obtained a global transcriptome of the apical meristem, revealed potential gene networks controlling the transformation from VM into IM in litchi [16]. In the present study, Genome-wide transcriptome analysis of the floral buds of the ‘Nuomici’ litchi trees under low and high-temperature conditions was carried out. We used ‘Nuomici’ as it is sensitive to HT in which floral buds easily cease to develop. Six RNA-Seq libraries were constructed, and 3.07–8.97 × 10^6^ clean reads were generated. To identify genes potentially involved in litchi floral abortion, we compared digital expression of the genes in the DP with that in the SP and identified 1320 up-regulated and 981 down-regulated DEGs. Then GO-term analysis was performed among the DEGs. In the biological process, we found that several temperature-, hormone-, and flowering-related terms were significantly enriched. Mainly basing on these terms, we identified flowering-related, hormone-related, and temperature responsive DEGs that might be involved in floral abortion.

Litchi panicle development undergoes inflorescence and floral organ development. These processes depend on floral meristem activity. The *ZHD4*, also called *FLORAL TRANSITION AT THE MERISTEM2* (*FTM2*), is expressed in the meristem and correlates with floral meristem growth in *Arabidopsis* [17]. In this study, microstructure observation of the floral buds indicated that floral meristem growth decreased or ceased under HT condition. Accordingly, the transcript level of the litchi homolog *ZHD4* was reduced, suggesting that *ZHD4* might be involved in the HT-induced floral abortion. Organ formation in plants is dependent on stem cell niches (SCNs) located in the meristems. Meristems show a functional zonation along the apical-basal axis and the radial axis. Organ primordia are formed in the circular peripheral zone (PZ) from stem cell descendants in which differentiation programs are activated. SCN actively suppresses stem cell identity in the PZ to allow organ development. In Arabidopsis, *AMP1* was proven to suppress ectopic SCN formation in the PZ [18]. ANT function in floral initiation and development, mutations in ANT, cause a decrease in floral organ number and alterations in the appearance of all floral organs [19]. *ANT* acts redundantly with *AP2* and function as a class A gene with regard to specification of petal identity [20]. The *AINTEGUMENTA-like* (*AIL*) genes are expressed primarily in actively dividing tissues. *AIL6* is expressed in inflorescence meristems and flowers [21]. ant ail6 mutants display a delay in the meristem identity transition and *LFY* induction. ANT and AIL6 transcription factors bind to the *LFY* promoter to control LFY mRNA accumulation, promote floral meristem identity transition [22]. In our litchi transcriptomic dataset, we identified *AMP1*, *ANT*, and *AIL6* whose expression was suppressed in HT compared to that in LT. It is suggested that they might be related to the cease of floral development under HT condition.

LHY is an essential clock component that plays an important role in photoperiodic flowering by controlling the rhythmic expression of flowering-time genes in *Arabidopsis* [23]. It repressed the floral transition under short-day and long-day conditions [24]. Our results showed that expression of the litchi homology *LcLHY* was higher in HT than that in LT. Accordingly, qRT-PCR analysis showed that *LcLHY* expression in inflorescence at HT was higher than that at LT in both the ‘Nuomici’ and ‘Huaizhi’. Contrarily, floral differentiation was repressed in HT. It is suggested that the increased expression in *LcLHY* might be related to the cease of floral development. It seems that floral abortion is regulated by a complicated gene network.

Plant hormones are signal molecules produced in plant cells and are expressed at extremely low concentrations but can regulate the formation of flowers, stems, leaves, control the development and ripening of fruit, and response to biotic and abiotic stresses [25]. Plant hormone levels are related to hormone homeostasis, biosynthesis, and the regulation of the biosynthetic process. In this study, we found that many hormone level regulated genes were differentially expressed between HT and LT conditions. *GH3.6* encodes an IAA-amino synthetase that prevents free IAA accumulation, maintains auxin homeostasis by conjugating excess IAA to amino acids [26]. *DIM* encodes Delta (24)-sterol reductase who plays a critical role in the general process of plant cell elongation, is involved in the conversion of 24-methylenecholesterol to campesterol, an early precursor of brassinolide [27]. *PAD4* encodes a lipase-like PAD4 participating in a positive regulatory loop that increases SA levels [28]. They all have higher expression levels in the SPs than in the DPs. In accordance with the gene expression profiles, it is suggested that HT treatment could change hormone homeostasis related gene expression, alter IAA, BR, and SA homeostasis conditions. These hormonal conditions might be related to the cease of floral development.

The plant hormones act as signals are transmitted to the nuclear by series signal transduction components to activate gene expression and result in physiological changes. Transcription factors are proteins that bind to a specific DNA sequence to control the transcription of specific genes. NAC, WRKY, and MYB transcription factors are central players in modulating transcriptional changes under hormonal control [29, 30]. In our RNA-seq dataset, we identified many *NAC*s, *MYB*s, and *WRKY*s from the enriched hormone related GO-terms. As the *NAC*s, *MYB*s, and *WRKY*s are the central players involved in the hormonal regulation, we hypothesized that they might play an important role in the HT-induced floral abortion. To quantify the correlation of these central players and the hormone related DEGs underlying floral abortion, we performed PLS-SEM analysis using the RNA-seq data and constructed a reliable model quantifying the relationship among hormone related DEGs, the *NAC*s, *MYB*s, *WRKY*s, and the flowering-related DEGs. Interestingly, the model showed that IAA related DEGs were connected with flowering-related DEGs indirectly while JA related DEGs made the contribution directly. Other hormone-related genes might affect it in both of the two pathways. On the whole, the effects of hormone-related DEGs on *NAC*s, *MYB*s, *WRKY*s were stronger than those on flowering-related DEGs, suggesting that the hormone related DEGs were more likely to affect flowering-related DEGs indirectly through *NAC*s, *MYB*s, and *WRKY*s. Hence *NAC*s, *MYB*s, *WRKY*s may act as central players involved in the HT-induced floral abortion underlying hormonal control. Using PLS-SEM analysis, we can figure out which one is more influential between the direct pathway and indirect pathways. Our study shows that it is possible to reveal the relationship between biological processes based on RNA-seq data using PLS-SEM.

From the enriched temperature responsive go term, we identify a homology *DREB1A*, encoding Dehydration-responsive Element-binding Protein. DREB1A is an APETALA2/ethylene-responsive element-binding factor (AP2/ERF)-type transcription factor. It specifically binds dehydration-responsive elements (DREs) to enhance late embryogenesis-abundant (LEA) protein levels and may accumulate under stress-induced dehydration in plant tissues [31–33]. *DREB1A* overexpression causes late flowering in plants [34]. In the present study, we found that expression of the *LcDREB1A* increased in the shrinking SP. Interestingly, a morphology comparison study on DPs and SPs showed that SPs might at last shrink to be stunt buds [4]. It seems that dehydration happens during this process. Hence, it is likely that *LcDREB1A* might be involved in the abortion of the floral buds in relation to dehydration.

To study detail expression patterns of the candidate genes in DPs and SPs, 12 genes identified from the flowering, hormone, and temperature response related DEGs were selected. The expression pattern of the inflorescence in ‘Nuomici’ and ‘Huaizhi’ at the four panicle developmental stages under LT and HT were studied. The results showed that *LcLHY*, *LcMED16*, *LcSKIP20*, *LcPOD20* in the two cultivars showed similar expression trends, negatively correlated with flowering. *LcAIL6* also had similar expression trends in the two cultivars, but positively correlated to flowering. Hence, it is suggested that increased transcript in *LcLHY*, *LcMED16*, *LcSKIP20*, *LcPOD20* and decreased transcript in *LcAIL6* might be related to the inhibition of floral development in ‘Nuomici’ and ‘Huaizhi’ under HT conditions. Other candidate genes showed a relationship with flowering in ‘Nuomici’ or ‘Huaizhi’, suggesting that our dataset is applicable for identifying genes involved in the HT-induced floral abortion in litchi.

Previous studies have proved that appropriate LT is needed for floral development in litchi [5, 6]. In accordance with these findings, our experiment performed under controlled environmental conditions also showed that the percentage of flowering terminal shoots of the LT-treated trees was significantly higher than that of the HT-treated trees, and that HT induced floral abortion and resulted in poor flowering. In open fields, warm winter and hot spring frequently happen due to climate change and global warming. Floral development of litchi may be inhibited by HT. In practice, control panicle leaf growth can reduce the HT-induced floral abortion by spraying with the ethylene producer ethephon [7], ROS inducer methyl viologen dichloride hydrate, and the NO donor sodium nitroprusside [5]. In the present studies, we have identified 127 plant hormone-related DEGs potentially involved in the HT-induced floral abortion. Interestingly, it has been proved that there is cross-talk between hormones and ROS/NO during plant growth and development [35]. Whether ethylene, ROS, and NO have any role in controlling the expression of these hormone-related DEGs and whether they inhibit floral abortion by the same mechanism as the LT does needs further detail investigation.

## Conclusion

Six RNA-Seq libraries of DP and SP were constructed, and 3.07–8.97 × 10^6^ clean reads were generated. Digital expression of the DPs with that of the SPs was compared, 1320 up-regulated and 981 down-regulated DEGs were identified. From the enriched GO-term, we identified 54 temperature responsive, 23 hormone homeostasis or biosynthesis related, 137 hormone signal transduction or responsive, and 18 flowering-related DEGs. It is suggested HT might affect flowering-related genes via transcription factors NACs, MYBs, and WRKYs through hormonal control. Increased transcript in *LcLHY*, *LcMED16*, *LcSKIP20*, *LcPOD20* and decreased transcript in *LcAIL6* might be related to the inhibition of floral development. Our studies provided potential genes for the future molecular breeding of new cultivars that can reduce floral abortion under warm climates, and a novel clue to reveal the relationship of biological processes based on the RNA-seq data using PLS-SEM.

## Methods

### Plant materials and experimental procedures

All the air-layered litchi trees (*Litchi chinenesis*) were cultivated in the experimental orchard of South China Agricultural University (lat. 23^o^9’40“N, long. 113^o^21’18”E). The air-layered seedlings were purchased from a market in Conghua District, Guangzhou. Litchi trees were planted in 30-L pots containing loam, mushroom cinder and coconut chaff (v: v: v, 3:1:1). The potted trees were subjected to LT for floral induction in open fields under winter condition.

For the identification of genes involved in floral abortion, four-year-old air-layered ‘Nuomici’ trees with similar size and phenological stage were selected. When panicle primordia emerged, eight trees were transferred to a growth chamber at 12-h photoperiod with a temperature of 18 °C (low temperature, LT) for 40 d to promote panicle development as controls. Another eight trees were transferred to a growth chamber at 12-h photoperiod with a temperature of 26 °C (high temperature, HT) for 2 weeks to induced abortion of panicles. Flowering conditions of the trees at HT or LT were calculated from eight replicated trees, respectively. Percentage of flowering terminal shoots was calculated as the percentage of the flowering terminal shoots to the total terminal shoots in one tree. The DPs under LT and the SPs under HT as shown in Fig. 1 c and d were collected for microstructure observation, or frozen in liquid nitrogen and stored at − 80 °C for RNA extraction and library construction. Each sample was collected from 2 to 3 trees. Three samples of the SP or DP were used for RNA extraction and library construction.

For the studies of floral development in different cultivars under low and high-temperature conditions, 6-year-old air-layered ‘Nuomici’ and ‘Huaizhi’ litchi trees were selected. Once panicle primordia emerged, five replicated trees of ‘Nuomici’, and five replicated trees of ‘Huaizhi’ were transferred to an LT growth chamber described above. Another five replicated trees of ‘Nuomici’, and five replicated trees of ‘Huaizhi’ were transferred to an HT growth chamber as controls. Percentage of flowering terminal shoots, percentage of leafy panicles, length of the panicles, length of the longest axillary panicles were determined. The inflorescences were collected at four stages according to Yang et al. [4]. These four stages under HT could be defined as primary stage, enlarging stage, shrinking stage, and shrunk stage. Those under LT could be defined as primary stage, enlarging stage, early elongating stage, and late elongating stage as shown in Fig. 7. At stage 1 under HT conditions (HT-S1), the axillary panicle primordia and the rudimentary leaves were at their primary stage of development. The axillary panicle primordia were just visible and the leaflets were conglutinated. At stage 2 (HT-S2), the axillary panicle primordia enlarged, the petiole of the rudimentary leaves began to elongate, and each individual leaflet could be seen. At stages 3 (HT-S3) and 4 (HT-S4), the petioles continued to elongate, and the leaflets began to expand, panicle primordia stopped developing. At stage 1 under LT condition (LT-S1), the axillary panicle primordia were just visible and the leaflets were conglutinated. At stage 2 (LT-S2), the axillary panicle primordia enlarged, the petiole of the rudimentary leaf elongated and each individual leaflet was possible to identify. At stage 3 (LT-S3), the axillary panicle primordia continue to enlarge while the petiole of the rudimentary leaves began to bend. At stage 4 (LT-S4), the axillary panicle primordia continued to enlarge and elongate, while the petiole of the rudimentary leaf continued to curve and could be abscised with a gentle touch. The samples under HT were collected every 8–10 d, while those at HT were collected every 2–3 d according to the developmental procedure. Samples were frozen in liquid nitrogen, stored at − 80 °C for total RNA extraction and gene expression analysis. Each sample of the SP or DP for gene expression analysis was collected from 1 to 2 trees.

### Light microscopy

Buds were vacuum penetrated, fixed with 4% polyformaldehyde for 4 h. Then 4% ethylenediamine was added to the buffer for tissue softening according to the method of Yang et al. [4]. The tissues were dehydrated in a graded ethanol series from 30 to 100% (*v*/v), with a final change to xylene, and then embedded in wax and sectioned in 8 μm using microtome (Leica RM2235, Nussloch, Germany). The tissues were stained with hematoxylin eosin and observed under a light microscope (Leica DMLB, Bensheim, Germany).

### RNA extraction, library construction, and RNA-sequencing

Total RNA extraction was carried out using Plant Total RNA Isolation kit (Huayueyang, Beijing, China). Oligo-dT beads (Qiagen, Valencia, USA) was used to enrich mRNA. The mRNA was fragmented into short fragments using fragmentation buffer, reversed transcribed into cDNA by random primers. Second-strand cDNA was synthesized using DNA polymerase I, RNase H, dNTPs. After that, the cDNA fragments were purified by Qiaquick PCR Purification Kits (Qiagen, Valencia, USA). The purified cDNA fragments were end repaired, added with poly (A), and ligated to Illumina sequencing adapters. The size selected fragments were amplified and sequenced by Illumina HiSeq™ 2500 at BGI (Shenzhen, China). A 50 single-end (SE) module was used. Six libraries including three biological replicates of DP and three of those of the SP were constructed (Table 1).

### RNA-Seq data analysis

Reads were mapped to the litchi transcriptome (http://litchidb.genomics.cn, unpublished) using Bowtie2 (version 2.1.0), with parameter settings followed the manual of eXpress (v1.5.1, https://pachterlab.github.io/eXpress/index.html). The Bowtie2 alignments were then subjected to eXpress for read counts and FPKM calculation of each transcript. All the raw data is available at the NCBI Short Read Archive (SRA) under the accession number PRJNA430479.

Differentially expressed genes (DEGs) between treatments were detected by DESeq2 [36], based on the ‘eff_counts’ matrices output from eXpress. Significant DEGs were restricted with FDR ≤ 0.01. GO term annotation of litchi transcriptome was obtained from a blastp search against the UniProtKB Swiss-Prot database (http://www.uniprot.org/uniprot/). The associated GO terms of the best hit target were assigned to each litchi transcript. The GO annotation was then used for GO term enrichment analysis of DEGs by the R Bioconductor package GOstats [37].

### Quantitative RT-PCR analysis

First-strand cDNA was synthetized using Reverse Transcriptase M-MLV (RNase H-) system (Takara, Dalian, China) from 1 μg extracted RNA. Quantitative real-time polymerase chain reaction (qRT-PCR) primers F1/R1 (Additional file 8: Table S4) were designed by Primer 6.0 (Premier Biosoft, Palo Alto, USA) and synthesized by Sangon Co. Ltd. (Shanghai, China). The litchi homolog *β-actin* was used as a reference gene (Additional file 8: Table S4). qRT-PCR was performed according to Lu et al. [9] on a LightCycler480 real-time PCR machine (Roche, Basel, Switzerland). The transcript quantification of the genes was performed in relation to the reference gene and they were calculated by 2^-△△CT^ method [38]. The analyses were conducted with three biological replicates and three technical replicates.

### Statistical analysis

Data were analyzed by SPSS (version 19.0; IBM Corp., Armonk, NY, USA). The differences among treatment means were evaluated by Duncan’s multiple range test at a 0.05 probability level.

To explore the relationships between DEGs and floral abortion, a hypothetical model according to Chen et al. [39] was specified and analyzed with PLS-SEM with the support of the SmartPLS 2.0 M3 software [40]. The standardized path coefficient values were generated with the PLS algorithm by Path Weighting Scheme using a bootstrapping method to obtain the significance of path coefficients; the Sign Changes were Individual Changes. The Samples during the calculation of bootstrapping method was 5000, and the Cases of ABA, BR, IAA, CTK, JA, SA related genes’ model was 88, 41, 38, 52, 56, 59 respectively.

## Additional files

Additional file 1: **Figure S1.** Correlation between qRT-PCR and RNA-seq. Scatter plots represent the fold-changes in the gene expression levels of SP compared to that of DP. (PDF 58 kb)

Additional file 2: **Figure S2.** Correlation analysis of the DPs and the SPs. (PDF 143 kb)

Additional file 3: **Table S1.** Ten top enriched GO terms of the DEGs in biological process (BP), cellular component (CC), and molecular function (MF) ontologies. (PDF 52 kb)

Additional file 4: **Figure S3.** Heat map diagram showing the up-regulated expression profiles of the hormone signaling related differentially expressed genes (DEGs) or hormone responsive DEGs. Litchi trees when panicle primordia emerged were transferred to a growth chamber at 12-h photoperiod with a temperature of 18 °C (LT) to encourage floral development. The other trees were transferred to a growth chamber at 12-h photoperiod with a temperature of 26 °C (HT) to induced floral abortion. FPKM values of the developing panicle (DP) and the shrinking panicle (SP) were normalized to Z-score. (PDF 952 kb)

Additional file 5: **Figure S4.** Heat map diagram showing the down-regulated expression profiles of the hormone signaling related and hormone responsive differentially expressed genes (DEGs). Litchi trees when panicle primordia emerged were transferred to a growth chamber at 12-h photoperiod with a temperature of 18 °C (LT) to encourage panicle development. The other trees were transferred to a growth chamber at 12-h photoperiod with a temperature of 26 °C (HT) to induced floral abortion. FPKM values of the developing panicle (DP) and the shrinking panicle (SP) were normalized to Z-score. (PDF 1712 kb)

Additional file 6: **Table S2.** Total effects and Bootstrapping analysis in PLS-SEM. T statistics higher than 1.96 were significant at 5% according to Hair et al. [15]. (PDF 51 kb)

Additional file 7: **Table S3.** PLS-SEM results quality criteria. All latent variables are significant and goodness-of-fit measures when Average variance extracted (AVE; Indicator for converge validity), and composite reliability (indicator for internal consistency reliability) are equal or higher than 0.5 and 0.7 according to Hair et al. [15]. (PDF 100 kb)

Additional file 8: **Table S4.** Primer sequences of the reference gene and candidate genes for qRT-PCR. (PDF 78 kb)

## Abbreviations

ABH: Cap-binding Protein Subunit
AGL: Agamous-like MADS-box protein
AMP: Glutamate Carboxypeptidase
ANT: AP2-like ethylene-responsive transcription factor
ATHB-7: Homeobox-leucine zipper protein
COL12: Zinc finger protein CONSTANS-LIKE 12
CRY: Cryptochrome
DEG: Differentially expressed gene
DP: Developing panicles
DREB1A: Dehydration-responsive element-binding protein 1A
ELF4: EARLY FLOWERING 4
HSFA2: Heat stress transcription factor A-2
HSP26-A: Probable glutathione S-transferase
HT: High temperature
LHY: Late Elongated Hypocotyls
LT: Low temperature
MED: Mediator of RNA Polymerase II Transcription Subunit
NO: Nitric oxide
PLS-SEM: Partial Least Squares Structural Equation Modeling
RNA-Seq: RNA-sequencing
ROS: Reactive oxygen species
RZ1A: Glycine-rich RNA-binding protein
SLD2: Delta (8)-fatty-acid desaturase 2
SP: Shrinking panicles
ZHD4: Zinc-finger homeodomain protein
