# Supplementary material for: Genome-wide transcriptome analysis reveals the molecular mechanism of high temperature-induced floral abortion in Litchi chinensis
Source: BMC Genomics. 2019 Feb 11;20:127. doi: 10.1186/s12864-019-5493-8 (PMC6371443; doi:10.1186/s12864-019-5493-8)
Supplement: Supplementary file 1 — Figure S1. Correlation between qRT-PCR and RNA-seq. Scatter plots represent the fold-changes in the gene expression levels of SP compared to that of DP. (PDF 58 kb) [file 12864_2019_5493_MOESM1_ESM.pdf]

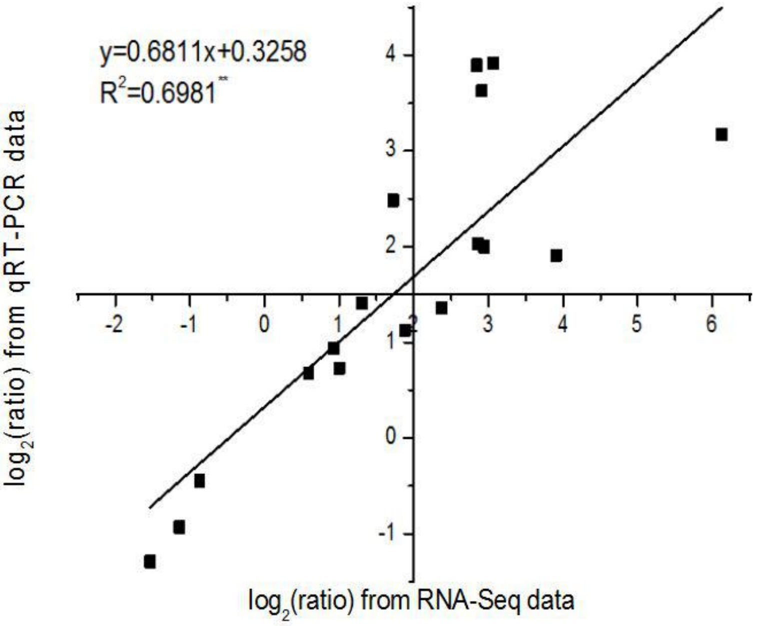

Figure S1. Correlation between qRT-PCR and RNA-seq. Scatter plots represent the fold-changes in the gene expression levels of SP compared to that of DP.
