# Supplementary figures and images for: Genome-wide transcriptome analysis reveals the molecular mechanism of high temperature-induced floral abortion in Litchi chinensis
Source: BMC Genomics. 2019 Feb 11;20:127. doi: 10.1186/s12864-019-5493-8 (PMC6371443; doi:10.1186/s12864-019-5493-8)

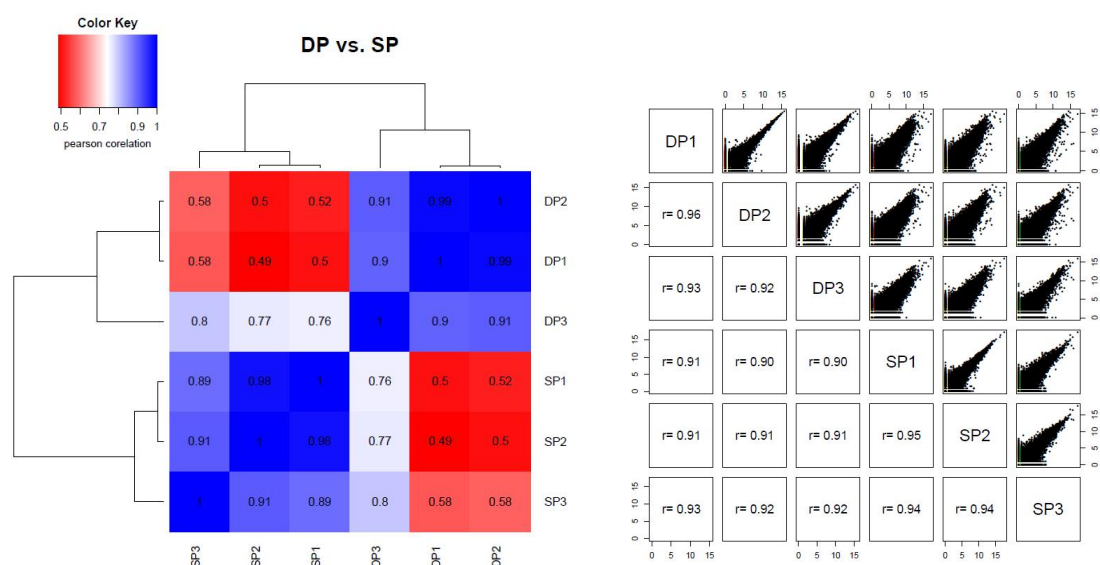

Figure S2. Correlation analysis of the DPs and the SPs.

Supplement: Supplementary file 2 — Figure S2. Correlation analysis of the DPs and the SPs. (PDF 143 kb) [file 12864_2019_5493_MOESM2_ESM.pdf]
