# Supplementary material for: Genome-wide transcriptome analysis reveals the molecular mechanism of high temperature-induced floral abortion in Litchi chinensis
Source: BMC Genomics. 2019 Feb 11;20:127. doi: 10.1186/s12864-019-5493-8 (PMC6371443; doi:10.1186/s12864-019-5493-8)
Supplement: Supplementary file 3 — Table S1. Ten top enriched GO terms of the DEGs in biological process (BP), cellular component (CC), and molecular function (MF) ontologies. (PDF 52 kb) [file 12864_2019_5493_MOESM3_ESM.pdf]

Table S1. Ten top enriched GO terms of the DEGs in biological process (BP), cellular component (CC), and molecular function (MF) ontologies.

Ontology	Term	No. of genes	Frequence	*P*-value
BP	Ribosome assembly	78	8.3%	0
BP	Mature ribosome assembly	92	9.8%	0
BP	Ribonucleo protein complex assembly	102	10.9%	0
BP	Ribonucleo protein complex subunit Organization	71	7.6%	0
BP	Ribosome biogenesis	91	9.7%	0
BP	Ribonucleoprotein complex biogenesis	97	10.4%	0
BP	Organelle assembly	100	10.7%	0
BP	Cellular macromolecular complex assembly	72	7.7%	0
BP	Macromolecular complex assembly	72	7.7%	0
BP	Macromolecular complex subunit organization	84	9.0%	0
CC	Cytosolic part	87	9.5%	0
CC	Cytosolic ribosome	80	8.7%	0
CC	Ribosomal subunit	79	8.6%	0
CC	Ribosome	82	8.9%	0
CC	Cytosolic large ribosomal subunit	50	5.4%	0
CC	Large ribosomal subunit	51	5.5%	0
CC	Intracellular ribonucleoprotein complex	116	12.6%	0
CC	Ribonucleoprotein complex	116	12.6%	0
CC	Non-membrane-bounded organelle	169	18.4%	0
CC	Intracellular non-membrane-bounded organelle	169	18.4%	0
MF	Structural constituent of ribosome	74	7.9%	0
MF	Structural molecule activity	80	8.5%	0
MF	Copper ion binding	21	2.2%	0
MF	Oxidoreductase activity	112	11.9%	0.0001
MF	Small ribosomal subunit rRNA binding	5	0.5%	0.0003
MF	Transition metal ion binding	34	3.6%	0.0003
MF	mRNA binding	37	3.9%	0.0004
MF	Nucleic acid binding transcription factor activity	134	14.3%	0.0005
MF	Large ribosomal subunit rRNA binding	4	0.4%	0.0007
MF	Transcription factor activity	132	14.1%	0.0009
