# Supplementary material for: Genome-wide transcriptome analysis reveals the molecular mechanism of high temperature-induced floral abortion in Litchi chinensis
Source: BMC Genomics. 2019 Feb 11;20:127. doi: 10.1186/s12864-019-5493-8 (PMC6371443; doi:10.1186/s12864-019-5493-8)
Supplement: Supplementary file 4 — Figure S3. Heat map diagram showing the up-regulated expression profiles of the hormone signaling related differentially expressed genes (DEGs) or hormone responsive DEGs. Litchi trees when panicle primordia emerged were transferred to a growth chamber at 12-h photoperiod with a temperature of 18 °C (LT) to encourage floral development. The other trees were transferred to a growth chamber at 12-h photoperiod with a temperature of 26 °C (HT) to induced floral abortion. FPKM values of the developing panicle (DP) and the shrinking panicle (SP) were normalized to Z-score. (PDF 952 kb) [file 12864_2019_5493_MOESM4_ESM.pdf]

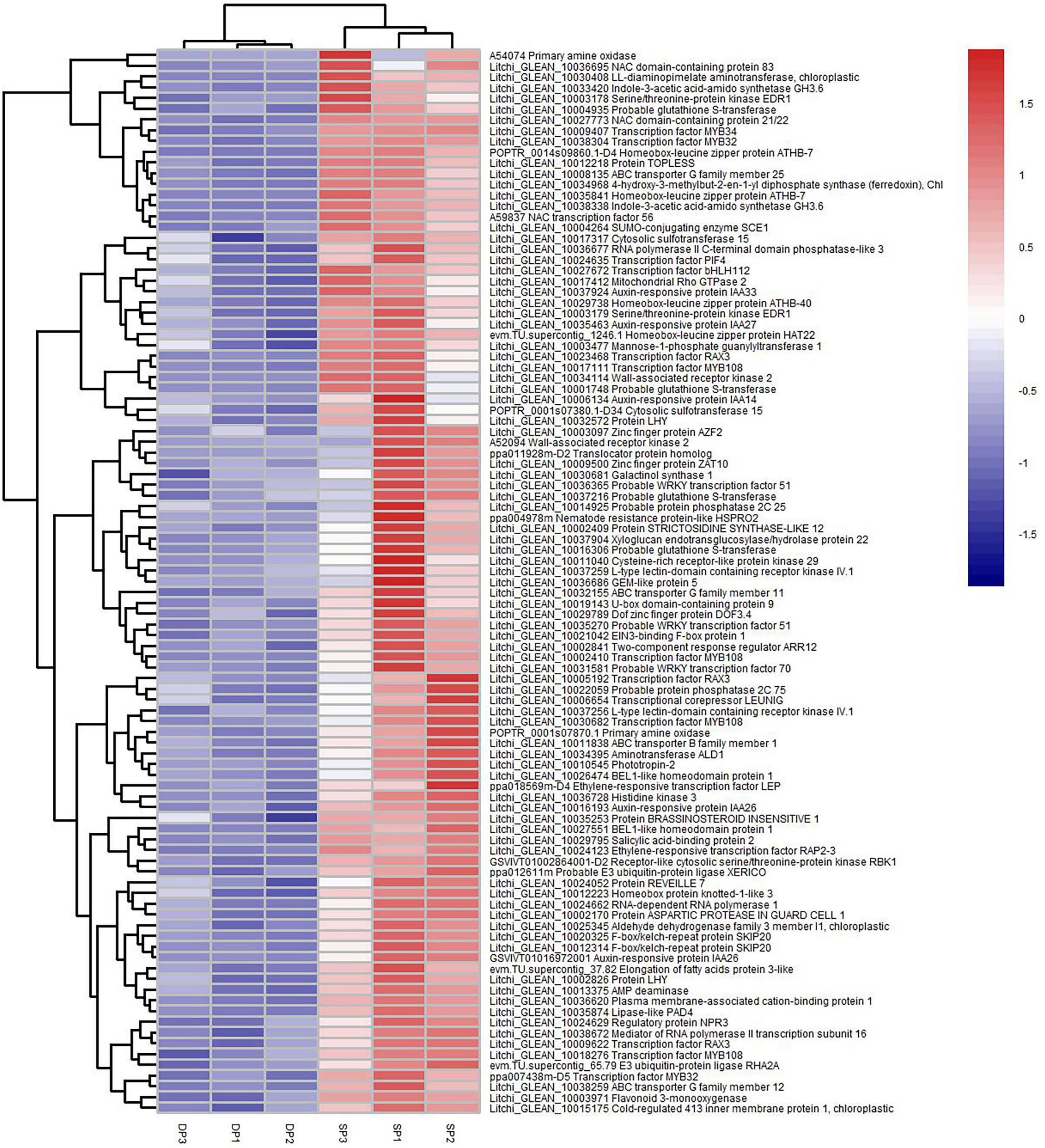

Figure S3. Heat map diagram showing the up-regulated expression profiles of the hormone signaling related differentially expressed genes (DEGs) or hormone responsive DEGs. Litchi trees when panicle primordia emerged were transferred to a growth chamber at 12-h photoperiod with a temperature of 18 ℃ (LT) to encourage floral development. The other trees were transferred to a growth chamber at 12-h photoperiod with a temperature of 26 ℃ (HT) to induced floral abortion. FPKM values of the developing panicle (DP) and the shrinking panicle (SP) were normalized to Z-score.
