# Supplementary material for: Genome-wide transcriptome analysis reveals the molecular mechanism of high temperature-induced floral abortion in Litchi chinensis
Source: BMC Genomics. 2019 Feb 11;20:127. doi: 10.1186/s12864-019-5493-8 (PMC6371443; doi:10.1186/s12864-019-5493-8)
Supplement: Supplementary file 6 — Table S2. Total effects and Bootstrapping analysis in PLS-SEM. T statistics higher than 1.96 were significant at 5% according to Hair et al. [15]. (PDF 51 kb) [file 12864_2019_5493_MOESM6_ESM.pdf]

Table S2 Total effects and Bootstrapping analysis in PLS-SEM. T statistics higher than 1.96 were significant at 5% according to Hair et al. [15].

Pathways during ABA modle	Original Sample (Total effects)	Sample Mean	Standard Deviation	Standard Error	T Statistics
ABA→MYB	0.995736	0.995779	0.000513	0.000513	1940.03006
ABA→NAC	0.920667	0.921755	0.009891	0.009891	93.081927
ABA→WRKY	0.947577	0.947885	0.008294	0.008294	114.252321
ABA→floral	-0.990627	-0.990719	0.001021	0.001021	969.958593
NAC→floral	-0.398866	-0.403003	0.054994	0.054994	7.252840
WRKY→floral	-0.108604	-0.113362	0.068071	0.068071	1.595457

Pathways during BR modle	Original Sample (Total effects)	Sample Mean	Standard Deviation	Standard Error	T Statistics
BR→MYB	-0.977255	-0.943359	0.256739	0.256739	3.806417
BR→NAC	-0.932888	-0.901132	0.247697	0.247697	3.766240
BR→WRKY	-0.904735	-0.873792	0.241443	0.241443	3.747203
BR→floral	0.987846	0.953367	0.259658	0.259658	3.804407
MYB→floral	0.507957	0.126344	8.276192	8.276192	0.061376
NAC→floral	-0.731007	-0.553788	4.425028	4.425028	0.165198
WRKY→floral	-0.595590	-0.373373	4.423844	4.423844	0.134632

Pathways during CTK modle	Original Sample (Total effects)	Sample Mean	Standard Deviation	Standard Error	T Statistics
CTK→MYB	0.993251	0.993360	0.001431	0.001431	693.854963
CTK→NAC	0.916780	0.918424	0.014509	0.014509	63.185847
CTK→WRKY	0.945395	0.946201	0.008931	0.008931	105.855240
CTK→floral	-0.990900	-0.990956	0.002091	0.002091	473.816329
MYB→floral	-0.358989	-0.659048	1.674963	1.674963	0.214327
NAC→floral	-0.208150	-0.068300	0.816072	0.816072	0.255063
WRKY→floral	0.130133	0.306254	0.977583	0.977583	0.133117

Pathways during IAA modle	Original Sample (Total effects)	Sample Mean	Standard Deviation	Standard Error	T Statistics
IAA→MYB	0.994018	0.994228	0.000708	0.000708	1403.05639
IAA→NAC	0.952943	0.954001	0.010494	0.010494	90.810788
IAA→WRKY	0.916738	0.918319	0.018270	0.018270	50.178145
IAA→floral	-0.995496	-0.995472	0.001276	0.001276	780.228056
MYB→floral	-0.705628	-0.703506	0.012329	0.012329	57.231170
NAC→floral	-0.308611	-0.310316	0.012422	0.012422	24.844923

Pathways during JA modle	Original Sample (Total effects)	Sample Mean	Standard Deviation	Standard Error	T Statistics
JA→MYB	0.996364	0.996441	0.000502	0.000502	1985.43711
JA→NAC	0.909458	0.911298	0.014208	0.014208	64.010417
JA→WRKY	0.957525	0.957769	0.009240	0.009240	103.632088
JA→floral	-0.987352	-0.987559	0.002001	0.002001	493.510641
NAC→floral	-0.337711	-0.339286	0.063057	0.063057	5.355670
WRKY→floral	0.039184	0.036587	0.088881	0.088881	0.440857

Pathways during SA modle	Original Sample (Total effects)	Sample Mean	Standard Deviation	Standard Error	T Statistics
SA→MYB	0.996364	0.996433	0.000486	0.000486	2048.08704
SA→NAC	0.909458	0.911052	0.013520	0.013520	67.267393
SA→WRKY	0.957525	0.957605	0.008823	0.008823	108.522342
SA→floral	-0.987352	-0.987526	0.001886	0.001886	523.576689
NAC→floral	-0.337711	-0.338393	0.060591	0.060591	5.573624
WRKY→floral	0.039184	0.037962	0.085700	0.085700	0.457222
