# Supplementary material for: Genome-wide transcriptome analysis reveals the molecular mechanism of high temperature-induced floral abortion in Litchi chinensis
Source: BMC Genomics. 2019 Feb 11;20:127. doi: 10.1186/s12864-019-5493-8 (PMC6371443; doi:10.1186/s12864-019-5493-8)
Supplement: Supplementary file 7 — Table S3. PLS-SEM results quality criteria. All latent variables are significant and goodness-of-fit measures when Average variance extracted (AVE; Indicator for converge validity), and composite reliability (indicator for internal consistency reliability) are equal or higher than 0.5 and 0.7 according to Hair et al. [15]. (PDF 100 kb) [file 12864_2019_5493_MOESM7_ESM.pdf]

Table S3. PLS-SEM results quality criteria.

quality criteria of ABA modle	AVE	Composite Reliability	R Square
ABA	0.820825	0.989303	
MYB	0.924885	0.988518	0.991491
NAC	0.934052	0.976994	0.847628
WRKY	0.968867	0.989402	0.897903
floral	0.885055	0.965987	0.997017

quality criteria of BR modle	AVE	Composite Reliability	R Square
BR	0.890041	0.030946	
MYB	0.924886	0.988519	0.955028
NAC	0.934167	0.977037	0.870279
WRKY	0.968854	0.989397	0.818545
floral	0.885058	0.965973	0.997854

quality criteria of CTK modle	AVE	Composite Reliability	R Square
CTK	0.886964	0.944659	
MYB	0.924888	0.988519	0.986547
NAC	0.934084	0.977006	0.840486
WRKY	0.968873	0.989404	0.893771
floral	0.885059	0.965963	0.998944

quality criteria of IAA modle	AVE	Composite Reliability	R Square
IAA	0.926261	0.651833	
MYB	0.924877	0.988517	0.988072
NAC	0.934151	0.977031	0.908100
WRKY	0.968858	0.989399	0.840408
floral	0.885047	0.966004	0.995177

quality criteria of JA modle	AVE	Composite Reliability	R Square
JA	0.876828	0.991027	
MYB	0.924888	0.988519	0.992740
NAC	0.934026	0.976984	0.827113
WRKY	0.968876	0.989405	0.916854
floral	0.885055	0.965984	0.997648

quality criteria of SA modle	AVE	Composite Reliability	R Square
SA	0.876828	0.991027	
MYB	0.924888	0.988519	0.992740
NAC	0.934026	0.976984	0.827113
WRKY	0.968876	0.989405	0.916854
floral	0.885055	0.965984	0.997648

All latent variables are significant and goodness-of-fit measures when Average variance extracted (AVE; Indicator for converge validity) and composite reliability (indicator for internal consistency reliability) are equal or higher than 0.5 and 0.7 according to Hair et al. [15].

Supplementary Table 4 Primer sequences of the reference gene and candidate genes for qRT-PCR.

Homology gene	gene ID	Sequence F（5′→3′）	Sequence R（5′→3′）
Actin		AGTTTGGTTGATGTGGGAGAC	TGGCTGAACCCGAGATGAT
AIL6	Litchi_GLEAN_10028656	ATGATGGTTCTGCGTCTG	AGTGGCTTTGGGTATGTG
LHY	Litchi_GLEAN_10002826	GAGGATAATGGCACAAGTAA	TGGGCTGAAATATGGAGT
CRY2	Litchi_GLEAN_10016315	AAGGGAAAGATAGCGTGAC	TCATTCCAGCATCTACCAA
MED16	Litchi_GLEAN_10038672	GCTGGGAAACAGTGATGT	GCTGAAGTCTGACGCAAA
WRKY70	Litchi_GLEAN_10031581	CATCCAGCACTCCATCAA	CTTCCAAAGGGACCAAAT
SKIP20	Litchi_GLEAN_10020325	CTGATGAGATAGCCATTGAGTGT	TGGGTTGATAATTGTTTAGGGAG
NAC100	ppa007653m	CAATCCTCCCTTACCCAATG	GCCCGAATCCTGAGAAACACT
NAC045	A41210	GATGCCAAGAAGACCAGG	AAGCGACAGACGACATAAGG
LAP	Litchi_GLEAN_10025024	AAGTTGAAAGTAACCTGCCTAG	AAGATTGCCGTCTCCTCC
POD4	Litchi_GLEAN_10019001	TTGTGATGGGTCAATACTTCT	AACATTCCAACTTGGTCCTC
POD53	Litchi_GLEAN_10031840	TGCTGGTCCAAACACGAA	GGCGGTCAAACTGTCCCT
MYB32	Litchi_GLEAN_10038304	ACAGCCCTCGCTTAGATC	AGCCTGAAGTGGGTAGTG
