# Supplementary material for: Genome-wide transcriptome analysis reveals the molecular mechanism of high temperature-induced floral abortion in Litchi chinensis
Source: BMC Genomics. 2019 Feb 11;20:127. doi: 10.1186/s12864-019-5493-8 (PMC6371443; doi:10.1186/s12864-019-5493-8)
Supplement: Supplementary file 8 — Table S4. Primer sequences of the reference gene and candidate genes for qRT-PCR. (PDF 78 kb) [file 12864_2019_5493_MOESM8_ESM.pdf]

Table S4. Primer sequences of the reference gene and candidate genes for qRT-PCR.

Homology gene	gene ID	Sequence F（5′→3′）	Sequence R（5′→3′）
Actin		AGTTTGGTTGATGTGGGAGAC	TGGCTGAACCCGAGATGAT
AIL6	Litchi_GLEAN_10028656	ATGATGGTTCTGCGTCTG	AGTGGCTTTGGGTATGTG
LHY	Litchi_GLEAN_10002826	GAGGATAATGGCACAAGTAA	TGGGCTGAAATATGGAGT
CRY2	Litchi_GLEAN_10016315	AAGGGAAAGATAGCGTGAC	TCATTCCAGCATCTACCAA
MED16	Litchi_GLEAN_10038672	GCTGGGAAACAGTGATGT	GCTGAAGTCTGACGCAAA
WRKY70	Litchi_GLEAN_10031581	CATCCAGCACTCCATCAA	CTTCCAAAGGGACCAAAT
SKIP20	Litchi_GLEAN_10020325	CTGATGAGATAGCCATTGAGTGT	TGGGTTGATAATTGTTTAGGGAG
NAC100	ppa007653m	CAATCCTCCCTTACCCAATG	GCCCGAATCCTGAGAAACACT
NAC045	A41210	GATGCCAAGAAGACCAGG	AAGCGACAGACGACATAAGG
LAP	Litchi_GLEAN_10025024	AAGTTGAAAGTAACCTGCCTAG	AAGATTGCCGTCTCCTCC
POD4	Litchi_GLEAN_10019001	TTGTGATGGGTCAATACTTCT	AACATTCCAACTTGGTCCTC
POD53	Litchi_GLEAN_10031840	TGCTGGTCCAAACACGAA	GGCGGTCAAACTGTCCCT
MYB32	Litchi_GLEAN_10038304	ACAGCCCTCGCTTAGATC	AGCCTGAAGTGGGTAGTG
